# Application of Ilmenite Mud Waste as an Addition to Concrete

**DOI:** 10.3390/ma13040866

**Published:** 2020-02-14

**Authors:** Filip Chyliński, Krzysztof Kuczyński, Paweł Łukowski

**Affiliations:** 1 Instytut Techniki Budowlanej, 00-611 Warsaw, Poland; k.kuczynski@itb.pl; 2 Warsaw University of Technology, Faculty of Civil Engineering, 00-637 Warsaw, Poland; p.lukowski@il.pw.edu.pl

**Keywords:** ilmenite mud, waste, concrete, titanium dioxide

## Abstract

Storing waste in concrete instead of landfills is environmentally friendly and also might make concrete more sustainable if some part is replaced with cement. This article presents a new way of valorising hazardous waste, namely ilmenite MUD from the production of titanium dioxide, which is used as a reactive additive to concrete. In fact, there are currently no articles presenting the way of valorisation that is presented in this paper. The global annual production of MUD is estimated to be about 0.7 million tons. Valorisation is possible due to the additional rinsing and filtering in the factory, which also confirms the novelty of this article. In this operation, the most hazardous compounds are returned back to the factory process. Rinsed mud (RMUD) is a pozzolanic reactive material with the potential use as a substitute of a part of Portland cement in concrete and other cementitious binders, like siliceous fly ash (FA). The level of RMUD pozzolanic activity is as high as the activity of siliceous fly ash. Comparative tests of concretes containing RMUD and fly ash, such as compressive strength, bending strength and shrinkage, were conducted. The concrete containing RMUD reached almost 90% of compressive and 108% of bending strength after 28 days of curing, compared to FA concrete. The results presented in this article are very promising and might point to a new way of valorising ilmenite mud waste.

## 1. Introduction

Concrete is increasingly used as a material in which to store waste instead of sending it to a landfill [1,2,3,4]. Paraphrasing Czarnecki [5], the issue is not that concrete needs more waste but that the environment should not accept any more waste in landfills. Concrete constructions have a projected life of at least 50 years and if we could recycle concrete and reuse it, as de Schepper et al. suggested [6], this timeframe could be several times longer. Also, the cement industry faces a problem meeting the growing demand for Portland cement because of the decreasing deposits of lime stone, slow increase of production and rising carbon taxes. A proposed way of dealing with this problem is using waste pozzolan instead of some part of the cement. [7] Ghaffar et al. [8] goes even further in ensuring the sustainable use of materials and the reduction in greenhouse gas emissions, and describes a circular construction concept, which is the idea of reusing materials from demolition for creating new constructions. Before waste can be reused, it has to be successfully used for the first time. The major problems in dealing with waste are the environmental safely of newly created composites and the influence on their durability [9] This article presents a way of valorising waste, namely ilmenite MUD from the production of titanium dioxide, which is used as a reactive additive for concrete.

Titanium dioxide (TiO_2_) is a white pigment, with a high refraction index and a high level of opacity, that is widely used all over the world mainly for the production of paints, plastics and paper. TiO_2_ is typically produced with one of two methods – chloride and sulphate. Each of these methods has its advantages and disadvantages. The chloride method generates far less waste products than the sulphate method, but for the production of TiO_2_ in the sulphate method, raw materials with lower concentration of TiO_2_ can be used. The global annual production of TiO_2_ in both methods is about 5.7 mln tons, of which about 35% is produced in the sulphate method. In Europe, the sulphate method is used for about 65% of production [10]. 

**Figure 1:**
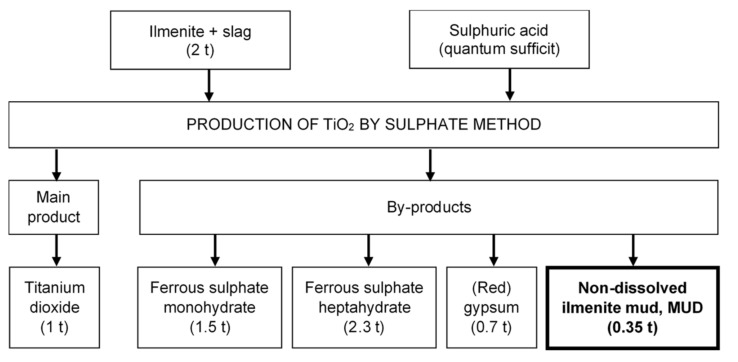
By-products generated per ton of the final product during the production of TiO_2_ using the sulphate method (data calculated according to a Spanish factory in Huelva [11,12,13]).

During the production of TiO_2_ in the sulphate method, a variety of by-products are produced alongside the main product (Figure 1), not all of which are useful. Some are treated as waste and placed in landfills. 

One of those so far useless materials is ilmenite mud (MUD), which is produced during the leaching of raw ilmenite with concentrated sulphuric acid. The leaching process, according to Reaction (1), can be described as:

FeTiO_3_ + 2H_2_SO_4_ → TiOSO_4_ + FeSO_4_ +2H_2_O. (1)

The main part of ilmenite is dissolved in acid but some remains, including all of the insoluble impurities from the ilmenite mineral. After separation of the solution, the liquid is carried to the next steps of the production, while mud remains. In most factories, MUD is categorised as a hazardous material according to European classifications [14] and is transported to special landfills. Sometimes it is neutralised before transport or mixed with other waste to lower its hazardous potential by decreasing the value of leaching of heavy metals and the concentration of sulphuric acid. The global annual production of MUD is estimated at about 0.7 mln tons [2]. 

In the literature, one can find suggestions for the possible valorisation of MUD waste. Potgieter et al. [15] attempted to use ilmenite mud waste for the production of Portland cement clinker by adding mud to the mixture of raw materials. They observed that the addition of mud at a level above 2% is detrimental to the properties of Portland cement, as it increases setting time and slows down the propagation of the cement’s compressive strength. The only observed advantage was that the addition of 1–2% of mud to the raw mixture lowered the temperature of fluxing. 

Gazquez et al. [12] used mud in the production of a fire-resistant material. The authors created a material containing mud that suited the requirements of fire-resistant materials at the level of the reference material. A problem was identified because the MUD they were using was classified as a NORM waste (Naturally Occurring Radioactive Material). This seems to be a main problem with increasing the content of MUD in tested materials. Another main disadvantage of this idea is that the valorisation of mud, as a constituent of fire-resistant materials, cannot use up all of the waste production. Contreraz et al. [16] and Garcia-Diaz et al. [17] used mud for the production of sulphur polymer concrete. They discovered that concretes containing up to 20% of mud had good mechanical properties compared to the reference. Heavy metals and radioactive nuclides were immobilised at a satisfactory level and their leaching was negligible. Ilmenite MUD can thus be used as a constituent of sulphur polymer concrete, although this type of mud valorisation has its limits because the relatively small production of this material is not enough to use up the entirety of the global production of ilmenite mud waste, or even a significant part of it. 

The most promising idea found in publications is the addition of waste to red ceramic, as described by Contreras et al. [18,19]. They discovered that the addition of mud to a raw mix at 3–10% had a beneficial effect on the sintering process. Also, they observed an increase of bending strength (up to 15%) and a reduction of porosity and water absorption (up to 50%). The possible amounts of waste, which can be used in this sector, make this idea the most promising of all those found in the literature.

This article presents a new way of valorising ilmenite mud waste as the authors did not find any similar publications. The idea was to use the waste as an additive to concrete. The global annual production of cement in the years 2014–2016 was calculated at about 4 bln tons [20], from which an estimated 30 billion tons of concrete were produced. Adding even a few percentiles of mud to a part of the globally produced concrete could solve the problem of the valorisation of this waste. The aim of this article was to show that using RMUD as a constituent of concrete can result in concrete at a similar level of usability as a concrete containing fly ash, which is a well-known and widely used additive in concrete production.

The high concentration of the remaining sulphuric acid (about 15%) makes this material useless for cement composites. Even after neutralisation with calcium hydroxide, the content of calcium sulphide is so high that it might increase setting time and lower the propagation of the compressive and bending strengths of cement. In order to counter these problems, a special batch of ilmenite mud waste was produced in the factory that has been additionally rinsed with water and filtered – rinsed mud (RMUD). As it was found, the leach contained some amounts of titanium sulphoxide, which is a very useful material for the production of titanium dioxide and, thus, it has returned to the production process. 

The process of rinsing mud decreased the content of sulphuric acid from about 15% to 1%. It also increased the percentage amount of silicon dioxide and aluminium oxide, and it lowered the content of most heavy metals. Heavy metals were immobilized in mortars that were not causing an environmental risk, and neither was the level of radioactivity of the raw RMUD [21]. All these aspects were very promising for the valorisation of waste as an additive to concrete. As also argued by Bobrowicz and Chyliński [22], RMUD has pozzolanic activity, which facilitates its addition to cement in order to create concrete or mortars.

This article presents the results of tests of concrete with the addition of RMUD compared to the same concrete containing siliceous fly ash in the place of RMUD. The following tests were made:

– consistency of fresh mix
– density of fresh mix
– compressive strength
– flexural strength
– water absorbability
– water permeability through concrete
– shrinkage
– scanning microscopy (SEM/BSE+EDS)

## 2. Materials and Methods 

### 2.1. RMUD, Fly Ash and Cement

**Figure 2:**
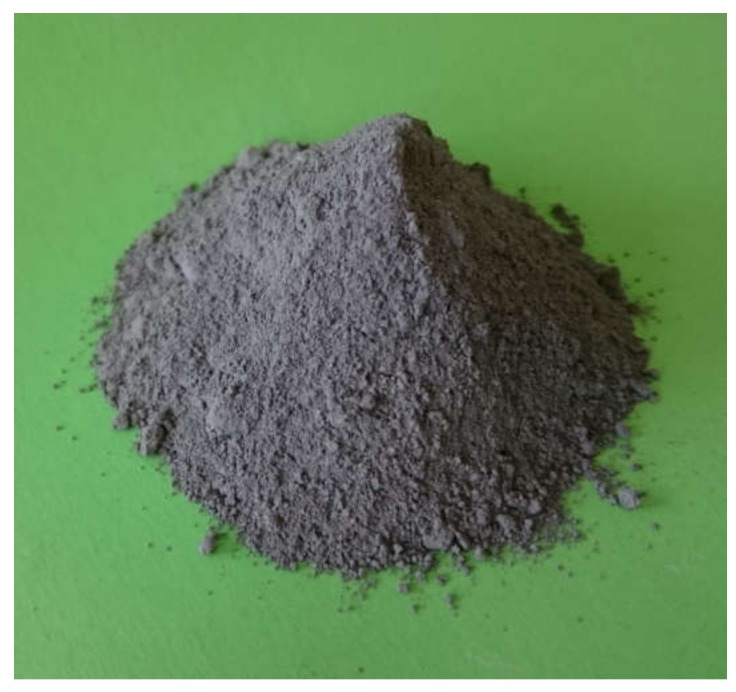
Sample of rinsed MUD (RMUD) prepared for tests.

After neutralisation of the remaining sulphuric acid with calcium oxide to a slightly acidic solution (pH value of water solution was about 5), RMUD was dried at a temperature of 105 °C. Then, it was sieved through a 0.5 mm sieve to discard larger conglomerates. Neutralisation to a slightly acidic pH aimed at stalling the start of a pozzolanic reaction of the material before it came into contact with the cement binder. Figure 2 presents what the prepared RMUD looked like.

As the reference concrete, siliceous fly ash was used according to the EN 450-1 standard [23]. Common Portland cement CEM I 42.5R according to the EN 197-1 standard [24] was used in the tests. Table 1 shows the concentrations of the main constituents in RMUD, siliceous fly ash and cement.

### 2.2. Concrete

Two types of concrete (“RMUD concrete” containing RMUD, and reference “FA concrete” containing fly ash) were prepared according to the EN 206 [25] standard for a class of aggression of XC1. This class of aggression specifies border parameters for concrete mixes as follows:

– minimum strength class C20/25
– minimum cement content 260 kg/m^3^
– maximum water binder ratio 0.65

**Figure 3:**
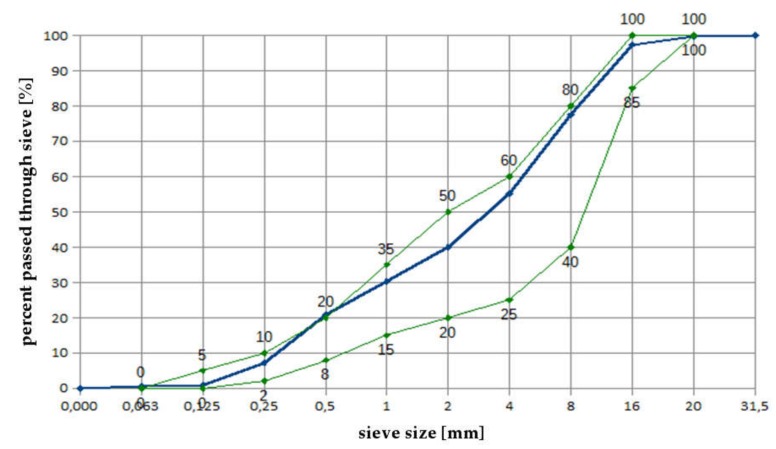
Sieving curves of aggregate mix used for concretes.

As aggregates, typical pebble gravel 2/8 and 8/16 coarse aggregate from central Poland, rinsed with mining sand, were used. Figure 3 shows a sieving curve of the prepared mixture of aggregates. Border curves are taken from the PN-B-06265 standard [26].

The amount of RMUD was 10.8% of the mass of the binder. This value was calculated from the statistical optimisation of mortars with the addition of RMUD [27]. Table 2 presents the composition of concretes. The quantity of cement was increased from 260 to 280 kg/m^3^ because, as the results of pre-tests showed, 260 kg of cement was not enough to reach the projected strength class on the 28th day.

### 2.3. Properties of Fresh Mix

The properties of the fresh mix, such as consistency, using slump loss according to the EN 12350-2 standard [28] and the density of the fresh mix according to the EN 12350-6 standard [29] were tested.

### 2.4. Compressive strength

Concrete cubes of 150 mm were formed according to EN 12350-1 [30]. After 28 and 90 days of curing in water at a temperature of 20 ± 2 °C according to the EN 12390-2 standard [31], compressive strength was tested according to EN 12390-3 [32]. Classes of compressive strength of concretes were calculated according to EN 206 [25], with criteria of acceptance for initial production.

### 2.5. Flexural Strength 

Samples of concrete with dimensions of 100 × 100 × 500 mm were formed. After demoulding, they were cured in water at a temperature of 20 ± 2 °C for 28 and 90 days. Flexural strength was tested according to EN 12390-5 [33]. Samples were loaded in two points. 

### 2.6. Absorbability of Concrete

The absorbability of concrete was tested according to PN-B-06250 [34]. Cubes of 100 mm after demoulding were cured in water for 90 days. After complete saturation, they were weighed and dried in an oven at a temperature of 105 °C for constant mass (± 0.2%).

### 2.7. Water Permeability Through Concrete

Water permeability through concrete was tested according to PN-B-06250 [34] on six 150 mm cubes for each concrete. The procedure was very similar to that described in EN 12390-8 [35]. The main difference was that PN-B allows us to test samples under a lower pressure than the obligatory 0.5 MPa. As it was feared that the test samples would not withstand such pressure, the pressure of water for this test was 0.4 MPa. After two days of water treatment, the samples were split and the depth of water penetration was measured. Samples were tested without using any hydrophobic impregnation like silanes or siloxanes or by adding any special additives that might enhance the concrete’s water impermability [36].

### 2.8. Shrinkage

**Figure 4:**
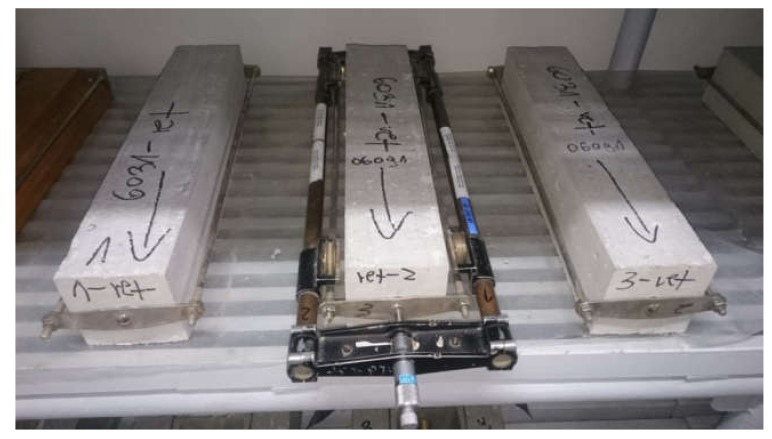
Measure of sample during shrinkage test.

Shrinkage tests were performed on 100 × 100 × 500 mm samples of concrete according to PN-B-06714-23 [37] using Amsler’s method. Three samples for each concrete were prepared. After demoulding, the samples were cured at a temperature of 20 ± 2 °C and humidity of 65 ± 5%. Samples were measured as shown in Figure 4 starting from the first day after demoulding until the 360th day.

### 2.9. Scanning Microscopy

For scanning microscopy observations, 100 mm concrete cubes were prepared. They were cured in water at a temperature of 20 ± 2 °C for 90 days. After that time, they were cut to sizes appropriate for microscopic analysis. After drying them at 40 °C, they were saturated with resin under a vacuum. After hardening, the resin samples were polished and vapour-deposited with gold.

Scanning electron microscopy (SEM) observations were carried out using the Leo SEM microscope (Carl Zeiss Microscopy GmbH, Köln, Germany) with a microscan EDS detector (Oxford Instruments, High Wycombe, UK). Secondary electrons (SE) and back-scattered electrons (BSE) images were collected. 

## 3. Results

### 3.1. Properties of Fresh Mix

Table 3 shows the results of fresh mix tests.

The tested properties, such as slump loss and density, of both mixed concrete containing RMUD and fly ash were almost identical. Differences between the density of RMUD (3.15 g/cm^3^) and fly ash (2.65 g/cm^3^) had no influence on the density of the fresh concrete mix. This was probably caused by the relatively small amount of additives to the mass of concrete. Another reason might be that the addition of RMUD caused a slight rise of air content, which is observed in mortars (Appendix A), and which lowers its influence on density. As Hill and Folliard [38] observed, the addition of fly ash to concrete may lower the air content in the concrete mix, which is caused mostly by unburned particles of coal in fly ash that might absorb the air particles.

### 3.2. Compressive Strength

**Figure 5:**
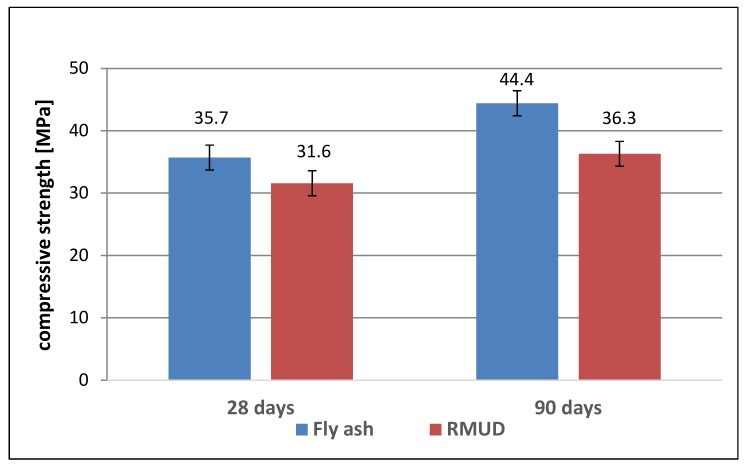
Compressive strength of concretes.

The results of the compressive strength of concretes are presented in Table 4 and Figure 5.

The class of concretes was calculated according to EN 206 for starting production. FA concrete reached one class higher than RMUD concrete after 28 and 90 days of curing. The differences between compressive strength at days 28 and 90 show the potential of pozzolanic activity of both additives because concretes, which were made using only CEM I as a binder (without additives), do not show such an increase, according to Gołaszewski et al. [39]. 

The compressive strength of FA concrete after 28 days was slightly higher than that of RMUD concrete. After 90 days, that difference had grown, although authors have proven similar levels of pozzolanic activity for RMUD and fly ash [22]. There are a few things that might explain the observed results. The first is the possibility that the air content in the RMUD concrete was higher, and the cement matrix was weaker than the reference. The results of tests with the addition of RMUD to mortars (Appendix A) show that increasing the amount of RMUD in mortar slightly increases the air content. A second explanation might be the effect of compaction. Fly ash has spherical grains with small diameters, which help in compacting the aggregate pile and might result in stronger binding forces than RMUD grains, which are sharp and irregular. 

### 3.3. Flexural Strength

**Figure 6:**
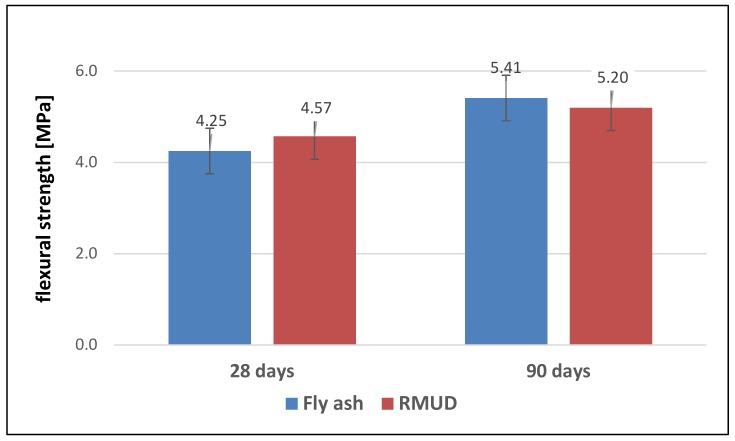
Flexural strength of concretes.

Figure 6 presents the results of flexural strength tests of FA concrete and RMUD concrete after 28 and 90 days. The values of flexural strength for both concretes were very similar

The differences of flexural strength for both concretes are smaller than the calculated uncertainty of the test method (± 0.5 MPa).

### 3.4. Water Absorbability of Concrete

**Figure 7:**
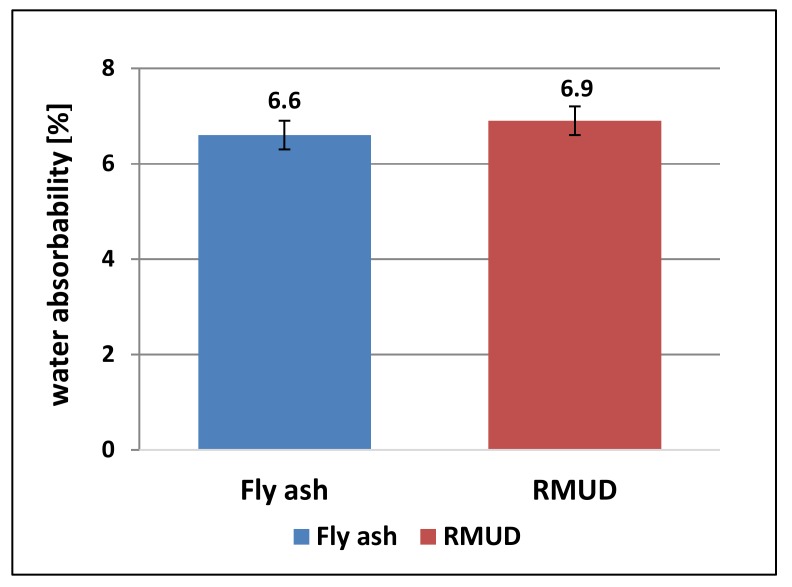
Water absorbability of concrete.

Figure 7 presents the results of the water absorbability test. Both concretes had almost the same absorbability—above 6%. This is quite a high value for concrete, but those concretes were not optimised for low absorbability, and the tests were carried out to spot differences between RMUD and FA concretes.

The differences of water absorbability for both concretes were smaller than the calculated uncertainty of the test method (± 0.3 MPa). The addition of about 10% of cement mass RMUD in the place of fly ash did not show any significant differences in water absorbability. Observed differences might be due to air entrained by the addition of RMUD. 

### 3.5. Water Permeability Through Concrete

**Figure 8:**
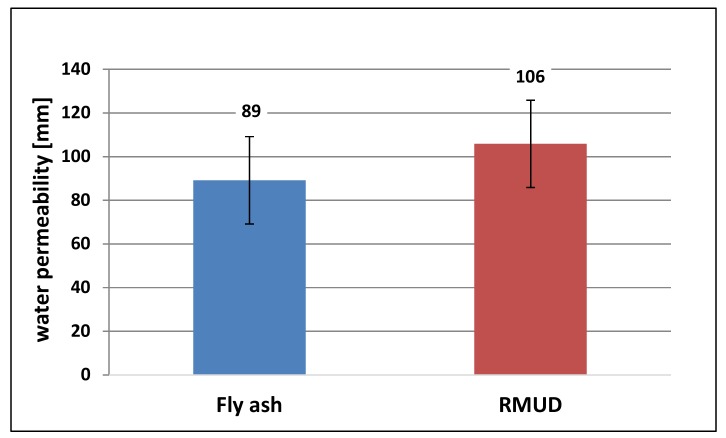
Average values of water permeability through concrete.

The results of water permeability through concrete are presented in Table 5 and Figure 8. The depth of water penetration under a pressure of 0.4 MPa was at the same level for both concretes. Penetration into RMUD concrete was a little deeper than in FA concrete, which conforms with the slightly higher results of absorbability. This shows that the structure of the RMUD concrete is a little bit more open than that of FA concrete, which also might be the effect of air entrained by the addition of RMUD. 

### 3.6. Shrinkage

**Figure 9:**
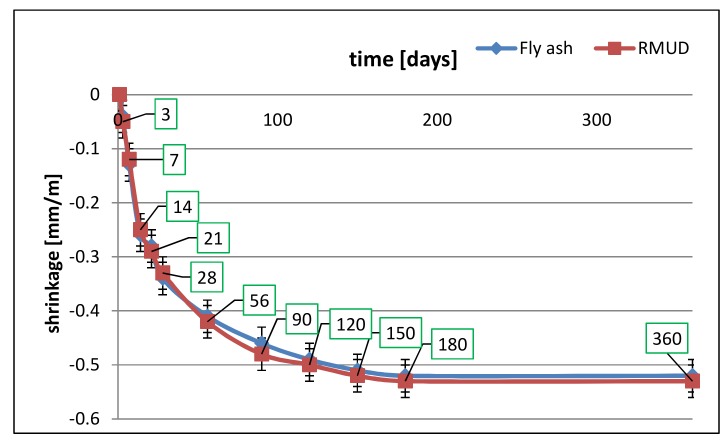
Results of shrinkage test.

Table 6 and Figure 9 present the results of shrinkage tests. 

The shrinkage of the RMUD concrete was almost the same as the FA concrete. The differences were well within the calculated range of uncertainty (± 0.03 mm/m). No effect of expansion was observed, which might indicate that small amounts of sulphuric acid from the waste do not affect sulphate corrosion of the concrete in these circumstances.

### 3.7. Scanning Microscopy

**Figure 10:**
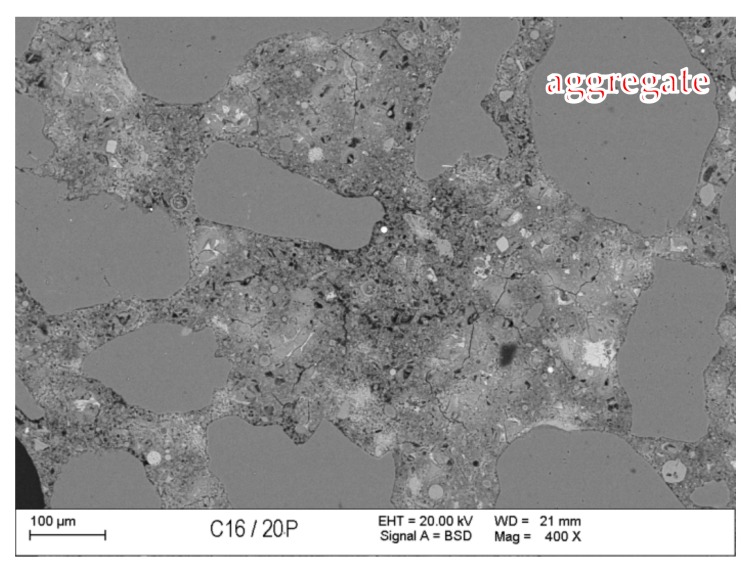
Back-scattered electrons (BSE) image of concrete with fly ash (FA).

**Figure 11:**
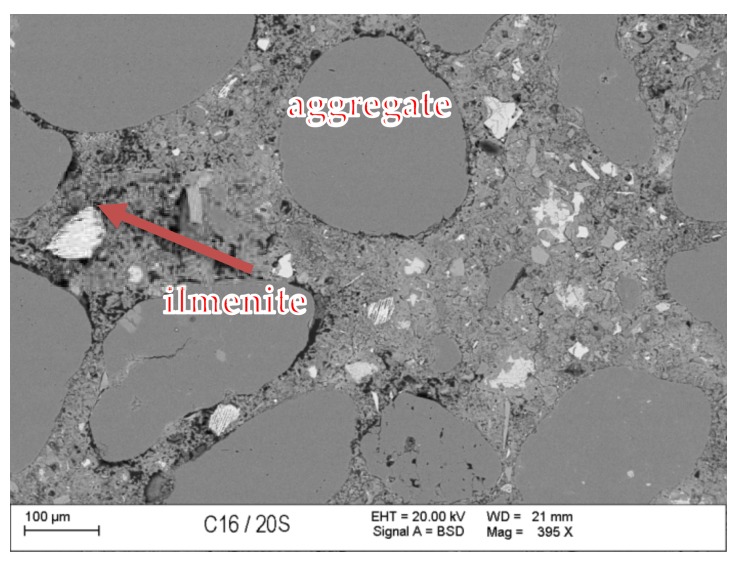
BSE image of RMUD concrete sample.

**Figure 12:**
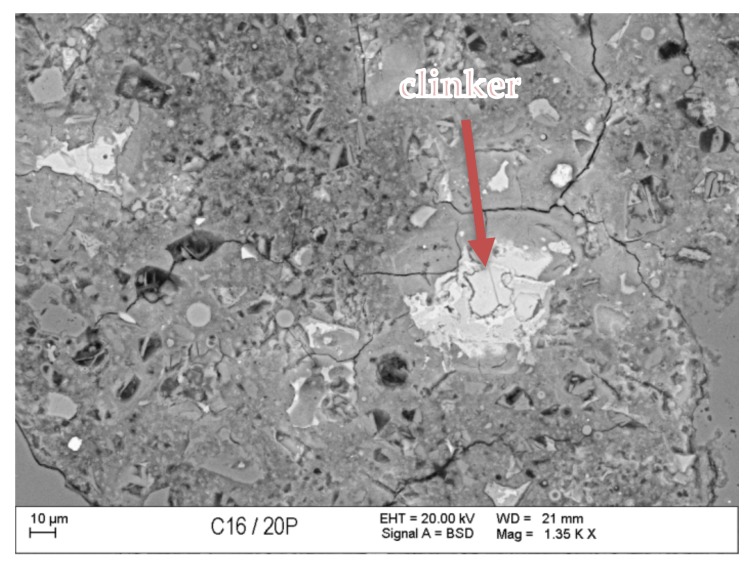
BSE image of FA concrete sample.

**Figure 13:**
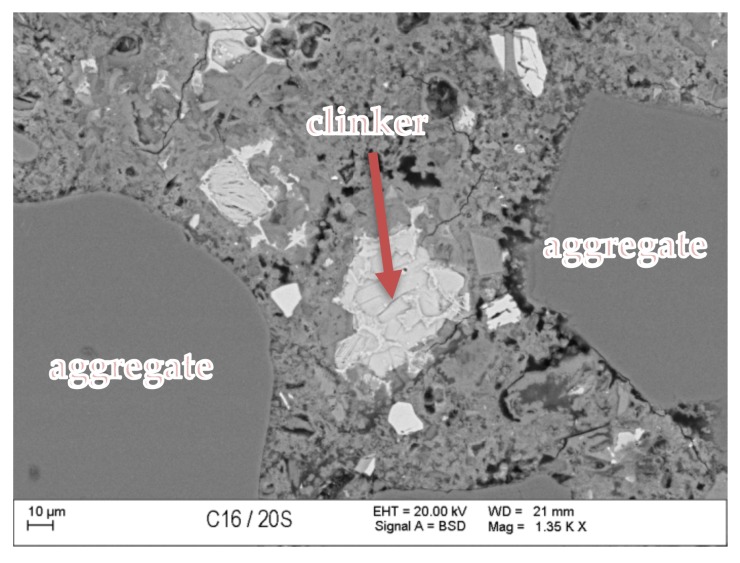
BSE image of RMUD concrete sample.

**Figure 14:**
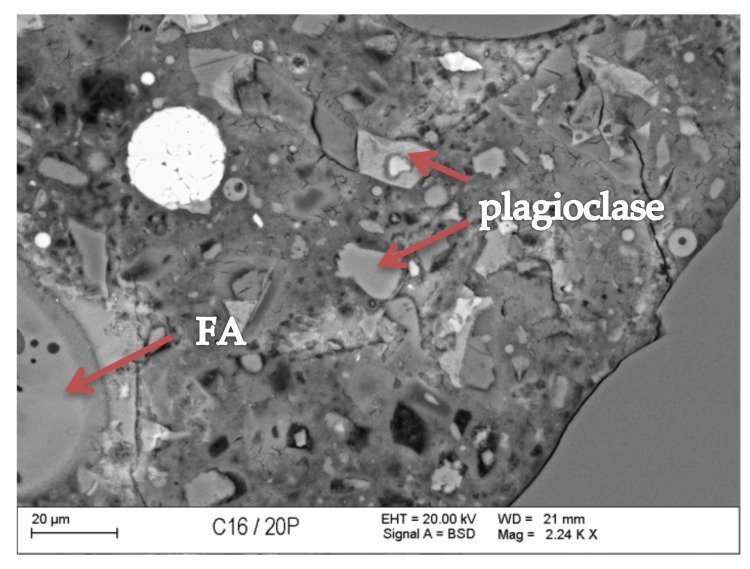
BSE image of FA concrete sample.

**Figure 15:**
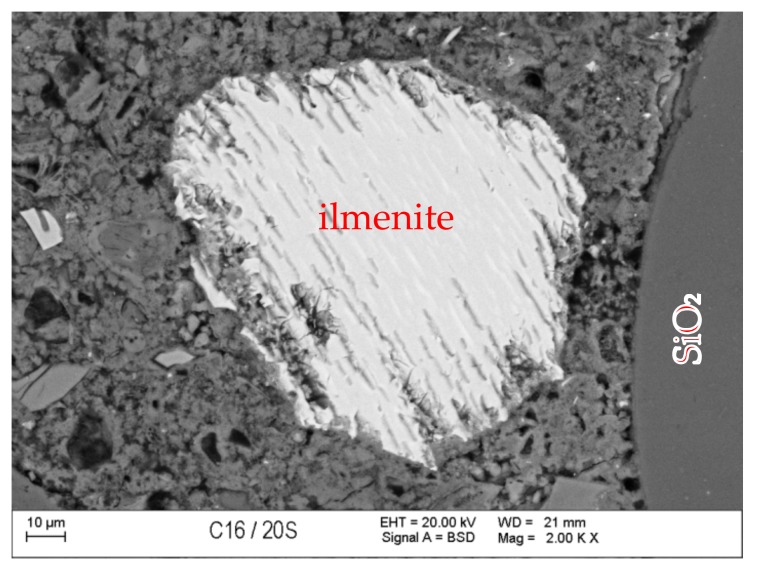
BSE image of RMUD concrete sample.

Figure 10, Figure 11, Figure 12, Figure 13, Figure 14 and Figure 15 present examples of SEM/BSE microscopic pictures of the surfaces of FA and RMUD concretes.

**Figure 16:**
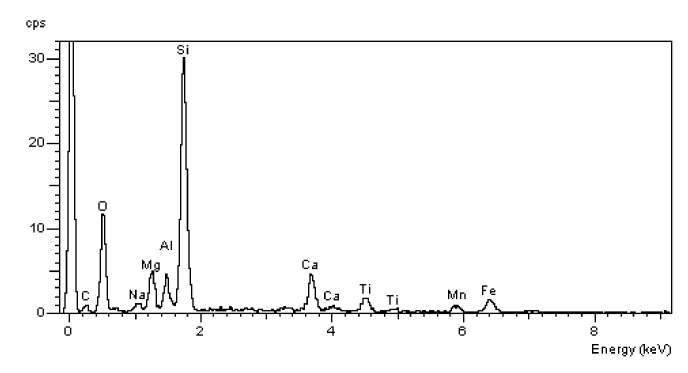
EDS spectrum of silicon dioxide glass phase.

FA samples were visibly less porous than RMUD ones. RMUD samples contained unreacted particles of waste such as: ruthyl, pyroxenes, plagioclases and slightly reacted silicon dioxide. Also, partly leached ilmenite grains with a CSH phase with a partly modified composition were observed between ilmenite layers. RMUD concretes contained an almost unreacted silicone dioxide glass phase with additions of other constituents, such as magnesium, aluminium, sodium calcium and traces of titanium iron and manganium (Figure 16). 

Both concretes contained CA and C_4_AF phases as relicts of the clinker phase. The CSH phase in RMUD and FA concrete was very similar and, in addition to calcium and silicon, also contained some amounts of aluminium, sulphur and, in some areas, traces of iron and titanium. A higher concentration of magnesium was observed in some parts of the CSH phase in RMUD concrete. The aggregate-grout zone was rich in portlandite.

## 4. Conclusions

Ilmenite mud waste from the production of titanium dioxide is a hazardous waste. As shown by the presented results, however, after some modifications, it might become a very useful material. Rinsing MUD with water helps to get rid of any remaining sulphuric acid and some of the heavy metals. The filtrate that is created during the process is also useful in further production of titanium dioxide. Thus, the process of rinsing does not generate any further waste. 

As shown by the test results, RMUD is a pozzolanic active material and might be used just like siliceous fly ash in cement composites. The addition of RMUD to concrete, as compared with that of fly ash, has no significant influence on the main properties of the fresh concrete mix (such as consistency or density). Its influence on compressive strength is quite similar to that of fly ash. The differences between compressive strength after 28 and 90 days of curing shows the potential activity of the material. Other publications [22] show clearly that, in fact, RMUD is a pozzolanic active material. Flexural strength is at the same level, taking into account the uncertainties of the test method. This proves that the addition of RMUD does not lower the strength of the cement binder. The absorbability of the concretes was at the same level and exceeded 6%. The higher specific surface of RMUD did not affect the sealing of the cement matrix. Water permeability through concrete containing RMUD was a little higher than fly ash. Both reached W4 level, according to the Polish standard PN-B-06250. Shrinkage tests did not show any expansion of concrete with RMUD compared to concrete containing fly ash. RMUD contains about 1% of sulphuric acid (neutralised to calcium sulphate) so it appears that those concretes are potentially less resistant to sulphate corrosion. It has to be borne in mind, however, that the addition of RMUD (or fly ash) replaces some part of cement. According to EN 197-1, cement should contain less than 3.0% of sulphates depending on the type.

The results of the tests performed did not show any disadvantages of using RMUD as a part of the binder in cement composites compared to fly ash. The following features has been observed in RMUD concrete compared to FA concrete:

– Slightly increase of consistency of fresh mix.
– No influence on density of fresh mix.
– Compressive strength reached 89% and 82% after 28 and 90 days of curing, respectively.
– Bending strength reached 108% and 96% after 28 and 90 days of curing, respectively.
– Water absorbability and water permeability were almost the same.
– Shrinkage during period of 360 days was almost the same.
– No structural defects were observed or any failures especially in zones surrounding grains of waste.

This study has shown that concrete might be successfully used as a store for this type of waste without it decreasing the concrete’s functional features. The most observed differences might be explained by the slight amount of air that entered the mix, which was caused by the addition RMUD to concrete. 

## Figures and Tables

**Table 1:** Concentration [%] of main constituents in RMUD, fly ash and cement.

	SiO_2_	TiO_2_	Fe_2_O_3_	MgO	Al_2_O_3_	CaO	Na_2_O	MnO	K_2_O	P_2_O_5_	SO_3_	Cl
RMUD	35.07	33.05	9.65	7.26	5.53	3.09	1.10	0.53	0.26	0.01	0.98	–
Fly ash	51.51	1.09	8.51	2.53	25.71	3.82	1.37	0.10	2.73	0.31	0.48	0.02
Cement CEM I 42.5R	20.06	–	3.38	0.89	4.13	64.41	0.24	–	0.56	–	2.97	0.07

**Table 2:** Composition of tested concretes.

Constituent	Quantity [kg/m^3^]
cement CEM I 42.5R	280
RMUD or fly ash	34 (10.8% b.m.)
aggregate 0/2 (rinsed mining sand)	838
aggregate 2/8 (pebble gravel)	516
aggregate 8/16 (pebble gravel)	573
water	204 (w/b = 0.65)

**Table 3:** Properties of concrete mix.

Property	FA Concrete	RMUD Concrete
slump loss [mm] (consistency class acc. to EN 206)	140 ± 10 (S3) *	150 ± 10 (S3) *
density of concrete mix [kg/m^3^]	2350 ± 20	2350 ± 20

* Class of consistency according to EN 206.

**Table 4:** Compressive strength of concretes.

Concrete	Compressive Strength f_ci_ [MPa]			Average Compressive Strength f_cm_ [MPa]	Standard Deviation [MPa] (Coefficient of Variation)	Compressive Strength Class acc. EN 206
FA 28 days	33.4	37.9	35.8	35.7 ± 2.0	2.3 (0.06)	C25/30
RMUD 28 days	31.5	31.8	31.5	31.6 ± 2.0	0.2 (0.01)	C20/25
FA 90 days	44.0	43.9	45.3	44.4 ± 2.0	0.8 (0.02)	C30/37
RMUD 90 days	37.6	34.5	36.7	36.3 ± 2.0	1.6 (0.04)	C25/30

**Table 5:** Water permeability through concrete.

Concrete	Depth of Water Penetration under Pressure of 0.4 MPa [mm]						Average [mm]	Standard Deviation [mm] (Coefficient of Variation)
FA	65	85	105	90	110	80	89 ± 20	17 (0.19)
RMUD	105	105	110	120	90	105	106 ± 20	10 (0.09)

**Table 6:** Results of shrinkage test.

Concrete	Average Shrinkage of Concrete [mm/m] Through Time [days]											
	1	3	7	14	21	28	56	90	120	150	180	360
FA	0.00	−0.04	−0.13	−0.26	−0.28	−0.34	−0.41	−0.46	−0.49	−0.51	−0.52	−0.52
RMUD	0.00	−0.05	−0.12	−0.25	−0.29	−0.33	−0.42	−0.48	−0.50	−0.52	−0.53	−0.53
	**Standard Deviation [mm/m]**											
FA	0.00	0.02	0.01	0.00	0.00	0.02	0.01	0.02	0.02	0.05	0.05	0.06
RMUD	0.00	0.01	0.02	0.01	0.01	0.01	0.02	0.02	0.02	0.03	0.03	0.03
	**Coefficient of Variation**											
FA	0.00	−0.50	−0.09	0.00	0.00	−0.06	−0.03	−0.04	−0.04	−0.08	−0.10	−0.11
RMUD	0.00	−0.25	−0.17	−0.05	−0.04	−0.03	−0.05	−0.04	−0.04	−0.06	−0.05	−0.06

**Figure A1:**
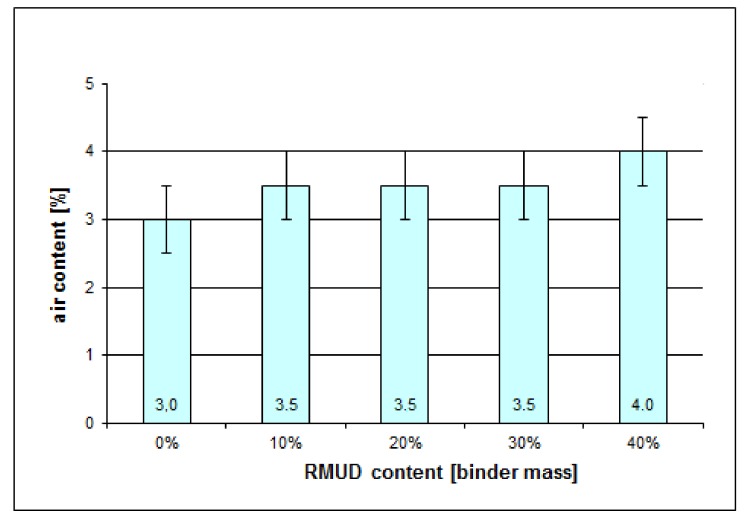
Influence of addition RMUD on air content in mortar.

## Appendix A

Table A1 and Figure A1 present an influence of addition RMUD on air content in mortar. Mortars were prepared according to EN 196-1 [40] and the air content was tested according to EN 1015-7 [41].

**Table A1:** Composition of tested mortars and air content.

Mortar	Composition [g]				Air Content
	RMUD	Cement	Sand	Water	
0%	0	450	1350	225	3.0 ± 0.5
10%	45	405	1350	225	3.5 ± 0.5
20%	90	360	1350	225	3.5 ± 0.5
30%	135	315	1350	225	3.5 ± 0.5
40%	180	270	1350	225	4.0 ± 0.5
